# The role of teacher-student developmental relationships in linking communicative language teaching and communicative competence

**DOI:** 10.3389/fpsyg.2025.1635428

**Published:** 2025-11-13

**Authors:** Danqiangyu Zhou, Qing Li

**Affiliations:** School of Foreign Languages, Civil Aviation Flight University of China, Guanghan, Sichuan, China

**Keywords:** communicative language teaching, communicative competence, teacher-students’ developmental relationships, Chinese English language learners, form-focused instruction

## Abstract

Interaction between educators and students plays a crucial role in shaping educational processes and outcomes. This study investigates the relationship between Communicative Language Teaching (CLT), Form-Focused Instruction (FFI), and communicative competence among Chinese English language learners, with a particular focus on the mediating role of teacher–student developmental relationships. Data were collected from 891 Chinese college students learning English as a foreign language. Results showed that CLT was positively associated with teacher–student developmental relationships, while FFI was negatively associated with them. Teacher–student developmental relationships, in turn, were strongly associated with students’ communicative competence. Importantly, CLT and FFI did not show significant direct effects on communicative competence, indicating that their influence operates indirectly through relational dynamics. These findings highlight the central role of teacher–student relationships in explaining how teaching approaches are linked with communicative competence. Given the cross-sectional design and the moderate reliability of some scales, results should be interpreted as exploratory. Future research with updated measurement tools, objective assessments, and longitudinal designs is needed to validate these associations.

## Introduction

The interplay between social and educational factors is central to many teaching–learning processes. Chinese-speaking learners of English as a foreign language encounter unique challenges due to linguistic, cultural, and educational differences between their native environment and English-speaking contexts (Chen et al., 2024; Savignon, 1987). These challenges shape language acquisition and highlight the need for teaching approaches that are linguistically effective yet sensitive to cultural and pedagogical realities (Im, 2025; Teng, 2023; Zhou, 2024).

Communicative Language Teaching (CLT) has long been promoted as a pedagogy that emphasizes authentic interaction, meaning negotiation, and functional language use in real-life contexts (Richards, 2005; Littlewood, 2014). In contrast, Form-Focused Instruction (FFI) deliberately directs attention to linguistic forms, either explicitly or incidentally (Long et al., 2001; Ellis, 2006). Rather than representing opposing paradigms, CLT and FFI embody complementary orientations that address fluency and accuracy, respectively. Their coexistence has become theoretically significant in second-language pedagogy because sustained communicative development depends on learners’ ability to integrate form-focused awareness with functional use (Spada, 2011; Larsen-Freeman, 2015). In the Chinese EFL context, however, the balance between these approaches remains contentious due to enduring exam-driven norms and the cultural premium placed on accuracy (Hu and McGrath, 2011; Jiang and Paulino, 2024; Zhu, 2025). Examining how these pedagogical orientations interact in practice is therefore crucial for understanding how Chinese teachers reconcile institutional expectations with communicative goals.

Teacher–student developmental relationships (TSDR) offer a relational lens through which these instructional tensions can be interpreted. Grounded in relational pedagogy (Noddings, 2005) and socio-constructivist theory (Vygotsky, 1978), developmental relationships emphasize trust, responsiveness, and mutual growth between teachers and students. Such relationships foster motivation, emotional security, and engagement—conditions that enhance learners’ willingness to communicate and their ability to transfer linguistic knowledge to authentic interaction (Riddle and Hickey, 2024; Scales et al., 2020). In EFL classrooms, especially in hierarchical or exam-oriented contexts like China, the quality of teacher–student relationships may determine how effectively CLT and FFI are implemented. A strong developmental relationship can humanize form-focused practices and sustain communicative learning within culturally constrained settings.

Research on CLT and FFI in China has expanded in recent years (Chen, 2024). Studies highlight both progress and persistent obstacles to implementation, such as limited teacher training, large class sizes, and strong emphasis on accuracy (Gu, 2025; Li and Xu, 2023; Xu and Wu, 2025). Other works show that localized adaptations of communicative or task-based approaches can enhance student outcomes in examination-oriented contexts (Lu et al., 2025). Despite these contributions, most studies focus either on pedagogical strategies or on communicative outcomes, leaving underexplored the relational processes that connect instructional practices with students’ learning experiences.

Teacher–student developmental relationships represent one such key process. They are known to foster motivation, emotional support, and active engagement, which in turn contribute to communicative competence (Riddle and Hickey, 2024). Recent Chinese evidence also indicates that strong teacher–student relationships can improve learners’ attitudes and cognitive performance (Ye and Wang, 2024). Yet, few studies have explicitly modeled how these relationships mediate the link between instructional approaches and communicative competence in Chinese EFL classrooms.

This study addresses three gaps: (1) the limited investigation of the mediating role of teacher–student developmental relationships in the CLT–FFI framework, (2) the need for culturally adapted pedagogical strategies to overcome the challenges faced by Chinese learners, and (3) the lack of context-specific evidence linking instructional approaches to communicative competence. By bridging these gaps, this study contributes to developing more effective and contextually appropriate instructional practices, offering insights for balancing communicative and form-focused instruction while strengthening relational dynamics in the classroom.

## Theoretical background

### CLT and FFI in Chinese English language classrooms

CLT emphasizes learners’ meaningful use of language in authentic social and cultural contexts, prioritizing communicative competence as the central instructional goal (Savignon, 1991; Richards, 2005). In contrast, FFI refers to pedagogical practices that deliberately direct learners’ attention to linguistic forms, especially grammar and vocabulary, either explicitly or incidentally (Long et al., 2001). Both approaches have been widely operationalized in empirical studies, particularly through the scales developed by Savignon and Wang (2003), which assess teachers’ and learners’ orientations toward communicative and form-focused instruction in Chinese EFL settings. Although the two approaches are conceptually distinct, they can also be complementary: CLT fosters fluency and interactional skills, whereas FFI helps consolidate accuracy within communicative tasks (Li and Xu, 2023; Xu and Wu, 2025). Importantly, the coexistence of these orientations can only be effective when mediated by the quality of teacher–student interactions, which determine how form-focused activities are perceived and integrated within communicative learning environments.

In China, traditional language education has historically prioritized grammatical accuracy and rote memorization, creating tension with the learner-centered, interactive practices inherent in CLT (Zhao, 2024; Zhu, 2025). Recent studies have underscored that teachers’ relational approaches—such as their responsiveness, encouragement, and emotional support—can help balance communicative and form-focused goals, thereby reducing the conflict between exam-oriented expectations and the principles of communicative pedagogy (Hu and Wang, 2023; Jiang and Paulino, 2024). This balance is particularly relevant for Chinese EFL learners, who typically achieve strong performance in standardized grammar assessments but often struggle with communicative confidence due to classroom anxiety and limited opportunities for authentic interaction (Gu, 2025; Liu and Liu, 2024).

Theoretically, while foundational frameworks such as Krashen’s Input Hypothesis (Krashen, 1982) and Canale and Swain’s communicative competence model (Canale and Swain, 1980) provided the basis for CLT, contemporary approaches increasingly emphasize psychological, social, and pedagogical dimensions. In this respect, teacher–student developmental relationships (TSDR) offer a socio-constructivist and relational pedagogy perspective (Vygotsky, 1978; Scales et al., 2020), highlighting that language learning is not only a cognitive but also a relational process. Through trust, feedback, and scaffolding, TSDR enable learners to internalize linguistic forms (supported by FFI) while maintaining authentic communicative engagement (central to CLT). For example, scaffolding techniques and teacher immediacy behaviors have been shown to reduce anxiety and enhance students’ willingness to communicate (Hu and Wang, 2023; Ye and Wang, 2024). Moreover, recent Chinese scholarship has expanded CLT research toward intercultural and digital communicative competence (Liu, 2025), underscoring the need for updated pedagogical frameworks that reflect the realities of globalized, technology-mediated learning environments (Yang and Yang, 2025; Sun, 2025).

Despite these advances, practical challenges persist. Large class sizes, exam-oriented curricula, and hierarchical teacher-centered traditions continue to constrain the adoption of communicative practices (Hagenauer et al., 2024; Zhu, 2025). Within such constraints, developmental relationships may serve as an enabling factor that allows teachers to humanize form-focused instruction and sustain communicative engagement, effectively bridging the gap between accuracy and fluency goals. Consequently, many teachers find it difficult to balance communicative interaction with traditional expectations, reinforcing the relevance of adaptive methodologies that combine communicative activities with explicit attention to linguistic forms.

In this study, we build on this body of literature by examining how teacher–student developmental relationships may mediate the effects of CLT and FFI on students’ communicative competence. By situating our model within contemporary empirical insights and recent Chinese research, we aim to clarify how relational dynamics intersect with instructional practices in shaping communicative outcomes in Chinese EFL classrooms.

### Communicative competence among Chinese English language learners

Communicative competence has become a central instructional objective for Chinese English language learners, especially as educational reforms progressively emphasize practical communication skills alongside traditional grammar-based instruction (Zhao, 2024). Building on Hymes’ seminal work (Hymes, 1973, 1983) and Canale (1987), whose multidimensional model underpins the present operationalization of communicative competence, communicative competence is now widely understood as a multidimensional construct that encompasses grammatical accuracy, sociolinguistic appropriateness, strategic language use, and discourse coherence in real-life communicative contexts (Guo et al., 2023; Jiang and Paulino, 2024).

While CLT prioritizes fluency and spontaneous interaction, and FFI directs learners’ attention to linguistic structures supporting accuracy, recent research emphasizes that these orientations yield their strongest effects when enacted through supportive teacher–student relationships (TSDR). Such relationships help learners internalize linguistic forms and apply them in meaningful communicative situations, thereby integrating accuracy with fluency (Scales et al., 2020; Riddle and Hickey, 2024). This relational mediation aligns with socio-constructivist views of learning (Vygotsky, 1978), in which teacher scaffolding, feedback, and emotional support create the psychological safety necessary for risk-taking and authentic communication. Consequently, communicative competence emerges not only from instructional input but also from the relational climate that sustains learners’ motivation and engagement.

At the same time, recent Chinese research highlights that communicative competence extends beyond linguistic proficiency to include intercultural and digital dimensions, reflecting the realities of globalized and technology-mediated communication. Studies conducted in Chinese high school and higher education contexts demonstrate growing attention to intercultural communicative competence (Zhou and Burhanudeen, 2023), as well as to innovative strategies that integrate cultural storytelling and digital platforms for language learning (Sun, 2025; Yang and Yang, 2025). These developments underscore that communicative competence in the Chinese EFL context involves not only accurate and fluent language use, but also the ability to navigate diverse cultural interactions and digital environments.

Despite this conceptual evolution, challenges persist in classroom practice. Many Chinese learners report high levels of classroom anxiety, limited opportunities for authentic communication, and a strong reliance on exam-oriented, grammar-centered instruction (Hu and Wang, 2023; Zhu, 2025). The presence of strong developmental teacher–student relationships can mitigate these barriers by promoting emotional security, increasing willingness to communicate, and helping students bridge the gap between structural knowledge and authentic performance (Ye and Wang, 2024). These barriers hinder the full development of communicative competence and highlight the need for adaptive pedagogies that balance fluency and accuracy while also fostering intercultural awareness and digital literacy.

In summary, communicative competence for Chinese English learners should be understood as a holistic construct that includes linguistic proficiency, interactive confidence, intercultural adaptability, and digital communicative ability. In the present study, communicative competence is examined as the key outcome variable within a model that links CLT and FFI practices through the mediating influence of TSDR, thereby clarifying how instructional and relational dimensions jointly shape learners’ communicative development.

### Dimensions of communicative competence among Chinese English language learners

Communicative competence is widely conceptualized as comprising multiple interrelated dimensions that enable learners to use language effectively in authentic contexts. The four classical components—grammatical, sociolinguistic, discourse, and strategic competence—remain foundational to communicative language ability (Canale, 1987; Guo et al., 2023), yet recent scholarship has expanded this framework to include intercultural and digital dimensions that reflect the realities of globalized, technology-mediated communication (Liu, 2025; Yang and Yang, 2025; Sun, 2025).

Grammatical competence involves mastery of linguistic structures such as syntax, morphology, and vocabulary. In the Chinese EFL context, grammar-oriented instruction traditionally ensures strong performance in this area but often limits spontaneous communication (Zhao, 2024). Sociolinguistic competence concerns the ability to use language appropriately across social and cultural contexts; explicit attention to intercultural norms has been shown to enhance learners’ adaptability and pragmatic awareness (Xu and Knijnik, 2024). Discourse competence relates to organizing speech and writing coherently, while strategic competence entails compensating for linguistic limitations through strategies such as paraphrasing, negotiation of meaning, and non-verbal cues (Guo et al., 2023). Finally, intercultural and digital competences extend these abilities by fostering culturally sensitive and multimodal communication practices essential to contemporary English use (Zhou and Burhanudeen, 2023; Yang and Yang, 2025).

From a pedagogical standpoint, CLT supports the development of fluency, interactional confidence, and discourse competence, whereas FFI contributes to grammatical accuracy and lexical precision. Their integration, when facilitated through supportive teacher–student developmental relationships (TSDR), allows learners to connect structural knowledge with authentic communication. Relational scaffolding, emotional support, and feedback not only enhance learners’ willingness to communicate but also strengthen strategic competence by encouraging risk-taking in interaction (Riddle and Hickey, 2024; Ye and Wang, 2024).

In summary, communicative competence among Chinese English learners should be understood as a holistic construct encompassing linguistic, sociocultural, and technological dimensions. Within this study, it serves as the outcome variable through which we examine how CLT and FFI practices—mediated by TSDR—jointly influence learners’ communicative development.

### Teacher–student developmental relationships as a mediator between communicative and form-focused instruction

Building on the concept of teacher–student developmental relationships (TSDR) introduced earlier, this section examines how such relationships mediate the effects of communicative and form-focused instruction on learners’ communicative competence. Multiple factors influence how teaching methods shape language outcomes, but the quality of teacher–student interactions has consistently been shown to affect students’ academic, emotional, and social development (Eddy et al., 2024). Following the conceptualization proposed by Scales et al. (2020), these relationships are characterized by trust, responsiveness, and mutual growth between teachers and students, forming a developmental bond that supports both motivation and learning. Grounded in socio-constructivist and relational pedagogy perspectives (Vygotsky, 1978; Noddings, 2005), developmental relationships create a supportive environment in which learners can benefit from both communicative and grammar-oriented practices. These relationships provide motivational and emotional scaffolding that encourages students to engage in communicative activities while persisting in structured linguistic tasks (Hagenauer et al., 2024; Jowett et al., 2023). This rationale supports the inclusion of TSDR as a mediator, given robust evidence linking it to language acquisition, motivation, and engagement (Liu and Liu, 2024; Yadav and Pandey, 2024).

Within CLT-oriented classrooms, positive developmental relationships foster an atmosphere of trust and reduced anxiety, encouraging learners to take communicative risks and engage in authentic exchanges. In FFI-based settings, supportive teacher feedback helps learners connect explicit knowledge of grammar and vocabulary with communicative use, thus integrating accuracy and fluency. Recent studies confirm that optimal communicative competence emerges when these instructional orientations are enacted in relationally supportive environments (Jiang and Paulino, 2024). Hence, TSDR can be viewed as the relational mechanism that humanizes form-focused practice and sustains communicative learning—an especially salient factor in hierarchical, exam-driven Chinese classrooms. Importantly, Chinese evidence also indicates that such relationships significantly contribute to learners’ motivation and cognitive development, even beyond language learning (Ye and Wang, 2024), underscoring their contextual relevance.

At the same time, it is important to acknowledge the methodological limitations of this study. Because the data are cross-sectional, mediation analyses can only suggest potential indirect pathways but cannot demonstrate causality or directionality. Following recommendations by Maxwell and Cole (2007) and Maxwell et al. (2011), our results are interpreted as exploratory rather than confirmatory. Future research using longitudinal or experimental designs will be necessary to test the causal mechanisms implied in the proposed model.

Given these theoretical considerations and the limitations of cross-sectional data, the following hypotheses were formulated for empirical testing:

Direct hypotheses:

H1: CLT is expected to be positively associated with teacher–student developmental relationships.
H2: FFI is expected to be positively associated with teacher–student developmental relationships.
H3: Teacher–student developmental relationships are expected to be positively associated with communicative competence.

Indirect hypotheses:

H4: Teacher–student developmental relationships mediate the association between CLT and communicative competence.
H5: Teacher–student developmental relationships mediate the association between FFI and communicative competence.

**Figure 1:**
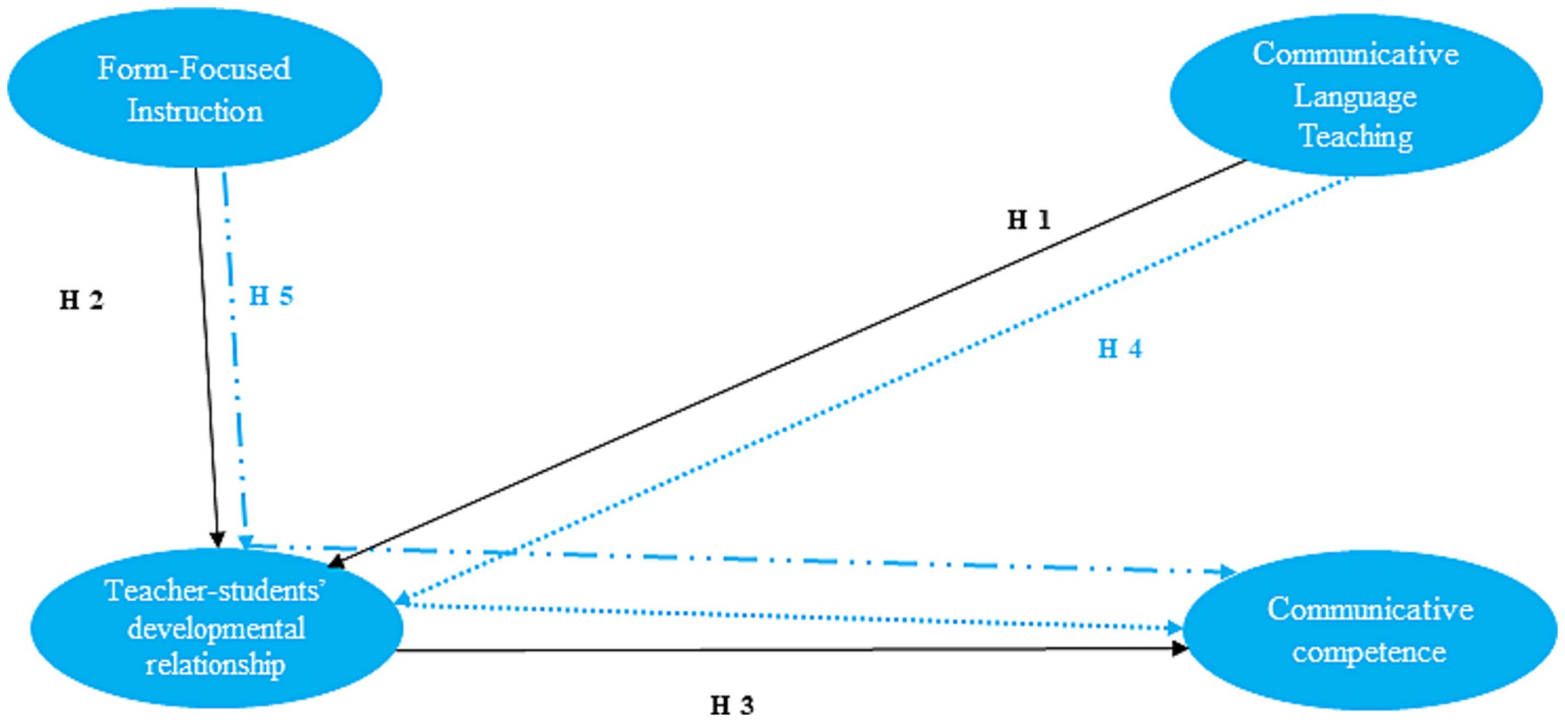
Research model with hypotheses. Blue dotted lines represent the indirect effects hypothesized in H4 and H5.

The research model is depicted in Figure 1.

## Method

### Participants

The study sample consisted of 891 Chinese college students enrolled in English as a Foreign Language (EFL) courses at public and private colleges and universities in Sichuan province. Participants ranged in age from 18 to 23 years (M = 20.78, SD = 1.69). The age distribution was as follows: 11.7% were 18 years old, 21.0% were 19, 7.2% were 20, 13.4% were 21, 31.2% were 22, and 15.6% were 23 years old. This distribution reflects the typical age range of undergraduate students in China and therefore ensures representativeness of the population under study.

Table 1 summarizes the demographic characteristics of the sample. Gender distribution was relatively balanced, with 42.6% male (*n* = 380) and 57.4% female (*n* = 511). Students came from a broad range of academic disciplines, with the largest proportion enrolled in education (34.3%), followed by sciences (15.3%), liberal arts (13.1%), agriculture (10.0%), engineering (7.9%), business and economics (4.9%), medicine (4.9%), and other fields (9.5%). This diversity strengthens the generalizability of the findings across different educational backgrounds.

**Table 1:** Demographic characteristics of participants (*N* = 891).

Variable	Category	n	%
Gender	Male	380	42.6
	Female	511	57.4
Age	18	104	11.7
	19	187	21.0
	20	64	7.2
	21	119	13.4
	22	278	31.2
	23	139	15.6
Discipline	Education	305	34.3
	Sciences	136	15.3
	Liberal arts	117	13.1
	Agriculture	89	10.0
	Engineering	70	7.9
	Business and economics	44	4.9
	Medicine	44	4.9
	Other	86	9.5

Age reported as percentages by year. M = 20.78, SD = 1.69.

Inclusion criteria were as follows: (1) enrollment as full-time college students in Sichuan province, (2) non-native English speakers learning EFL as part of their college curriculum, (3) aged between 18 and 23 years, and (4) provision of informed consent prior to participation. Participants were recruited through convenience sampling via social networks, which enabled us to capture a diverse sample across multiple institutions and disciplines.

### Procedure

The study adhered strictly to ethical guidelines and fully complied with the principles outlined in the Declaration of Helsinki for research involving human participants. Before data collection, ethical approval was obtained from the Institutional Review Board of Civil Aviation Flight University of China, with approval number IRB-2024-078 granted on January 15, 2024. All participants provided informed consent, ensuring they were fully aware of the study’s purpose, voluntary participation, and right to withdraw without any negative consequences. Confidentiality of their data was guaranteed throughout the study.

To ensure transparency and consistency in the data collection process, we employed a standardized online questionnaire through the Questionnaire Star platform. The survey link was distributed via widely used social networks, including WeChat and QQ, targeting EFL students across public and private institutions.

The survey included demographic questions and standardized scales measuring CLT, form-focused instruction, teacher-students’ developmental relationships, and communicative competence. Participants completed the questionnaire online at their convenience, reducing potential biases associated with in-person data collection. To minimize nonresponse bias, reminders were sent periodically to ensure active participation and completion within the data collection window. All instruments were administered in English, as participants were upper-year English majors. Although no formal back-translation or piloting was conducted, comprehension was verified before data collection, and clarifications were provided in Chinese when necessary to ensure understanding. No reports of item misunderstanding were recorded.

Data collection occurred between February 1, 2024, and March 15, 2024. The web-based approach allowed for broad geographic coverage across Sichuan province and ensured accessibility for all participants, including those from rural or remote areas. This approach gave participants flexibility while maintaining data integrity and ethical research standards.

### Instruments

#### Communicative language teaching (CLT)

Participants’ perceptions of CLT practices were measured using items adapted from Savignon and Wang (2003). This scale assessed the extent to which classroom activities emphasized authentic communication, interaction, and opportunities to use English meaningfully. Sample items included:

- “My English teachers often designed activities for us to interact in English with peers.”
- “Our focus in class was communication, but the teacher would explain grammar when necessary.”
- “My English teachers often created an atmosphere for us to use English.”

The Cronbach’s alpha for this scale was 0.65, indicating moderate internal consistency. Although below the conventional threshold of 0.70, we retained the scale because it captures the multidimensional nature of CLT practices in the Chinese context, where implementation often varies across institutions.

#### Form-focused instruction (FFI)

Perceptions of FFI were assessed with items also adapted from Savignon and Wang (2003). This scale measured the extent to which instruction prioritized grammar explanations, drills, and teacher-centered practices. Sample items included:

- “English teaching in my college was grammar-focused.”
- “My English teachers often asked us to do sentence drilling and repeat sentences after them.”
- “English teaching in my college mainly explained and practiced grammar rules.”

The Cronbach’s alpha for the FFI scale was 0.68, again reflecting moderate internal consistency. Despite not reaching the preferred reliability standard, the scale provides valuable insight into how students perceived accuracy-oriented practices in their classrooms.

We acknowledge that neither scale fully captures the richness of CLT and FFI as implemented in Chinese classrooms. In particular, more authentic features of CLT such as role-plays, group projects, and negotiation of meaning were not systematically included in the present items. We note this as a limitation of the current study. Future research should develop and pilot revised instruments with stronger psychometric properties (*α* ≥ 0.70) and a broader representation of instructional practices, in order to more accurately distinguish between communicative and form-focused approaches in Chinese EFL contexts.

#### Teacher–student developmental relationships (TSDR)

Teacher–student developmental relationships were measured using a five-item scale adapted from Scales et al. (2020), which captures key dimensions such as expressing care, challenging growth, providing support, sharing power, and expanding possibilities. Sample items included:

- “My teachers listen to me when I talk” (Express care).
- “My teachers have high expectations for me” (Challenge growth).
- “When I have a problem at school, my teachers help me figure out who I should talk to for help” (Provide support).
- “My teachers take time to consider my ideas when making decisions” (Share power).
- “My teachers help me discover new things that interest me” (Expand possibilities).

The Cronbach’s alpha for this scale was 0.74, indicating adequate reliability. This instrument has been validated in previous studies and provides a multidimensional yet concise measure of the quality of developmental relationships in educational contexts.

#### Communicative competence (CC)

Communicative competence was assessed with a four-item scale adapted from Canale’s (1987) multidimensional framework, covering grammatical, sociolinguistic, discourse, and strategic competence. Sample items included:

- “I can use English grammar correctly when forming sentences in both speaking and writing” (Grammatical competence).
- “I understand how to adjust my language use depending on whether I am speaking to a teacher, a peer, or in a formal situation” (Sociolinguistic competence).
- “I can organize my ideas clearly in English when writing essays or giving presentations” (Discourse competence).
- “When I forget a word in English, I can use other words or gestures to express my meaning” (Strategic competence).

The Communicative Competence Scale was adapted from Canale (1987), encompassing the four classical dimensions of grammatical, sociolinguistic, discourse, and strategic competence. This operationalization was selected to maintain conceptual alignment with the theoretical model and comparability with prior EFL studies conducted in China. Although more recent frameworks have expanded the construct to include intercultural and digital dimensions (e.g., Zhou and Burhanudeen, 2023), the classical structure remains a validated and widely adopted foundation for empirical research in communicative language teaching. The present CFA results confirmed that this instrument demonstrated strong internal consistency (*α* = 0.85), satisfactory composite reliability (CR = 0.87), and acceptable convergent and discriminant validity indices (see Table 2). These findings indicate that the scale provides a reliable and theoretically coherent measure of communicative competence within the present research scope, while future studies may consider adopting updated multidimensional frameworks as the field evolves.

**Table 2:** Reliability, convergent validity, and discriminant validity of the constructs.

Construct	α	CR	AVE	√AVE	MSV	ASV	Fornell–Larcker
Communicative language teaching (CLT)	0.65	0.74	0.66	0.81	0.40	0.27	Satisfied
Form-focused instruction (FFI)	0.68	0.63	0.36	0.60	0.35	0.29	Satisfied
Teacher–student developmental relationships (TSDR)	0.74	0.74	0.42	0.65	0.34	0.28	Satisfied
Communicative competence (CC)	0.85	0.87	0.63	0.79	0.34	0.29	Satisfied

α, Cronbach’s alpha; CR, Composite Reliability; AVE, Average Variance Extracted; MSV, Maximum Shared Variance; ASV = Average Shared Variance. All HTMT ratios were below 0.85, indicating discriminant validity across constructs.

#### Reliability and validity of the instruments

To evaluate the reliability and validity of all measurement instruments, a confirmatory factor analysis (CFA) was conducted in AMOS 29 prior to testing the structural model. All standardized factor loadings were statistically significant (*p* < 0.01) and above the minimum recommended threshold of 0.50, confirming that each observed indicator represented its respective latent construct. The results indicated that the measurement model exhibited satisfactory psychometric properties across all variables.

Cronbach’s alpha coefficients ranged from 0.65 to 0.85, suggesting moderate to high internal consistency. Composite reliability (CR) values ranged from 0.63 to 0.87, all exceeding the recommended cutoff of 0.60 (Hair et al., 2021). The average variance extracted (AVE) ranged from 0.36 to 0.66, reflecting adequate convergent validity for most constructs. Although the AVE values for the Form-Focused Instruction (FFI) and Teacher–Student Developmental Relationships (TSDR) scales were slightly below the ideal threshold of 0.50, their composite reliability values were above 0.70, supporting acceptable construct reliability (Fornell and Larcker, 1981). Given that all indicators loaded significantly on their intended factors, the instruments can be considered psychometrically sound for exploratory modeling in this educational context.

Discriminant validity was assessed using both the Fornell–Larcker criterion and the Heterotrait–Monotrait (HTMT) ratio. For each construct, the square root of the AVE exceeded the interconstruct correlations, thereby satisfying the Fornell–Larcker criterion. In addition, all HTMT ratios were below 0.85, confirming that the constructs were empirically distinct from one another (Henseler et al., 2015). These results provide robust evidence that the measurement model demonstrates both convergent and discriminant validity.

Table 2 presents the main indices of reliability, convergent validity, and discriminant validity for the four constructs included in the study.

#### Analyses procedures

Data analysis proceeded in two stages. First, descriptive statistics and Pearson correlations were computed using SPSS to summarize sample characteristics and examine initial associations between the study variables (CLT, FFI, teacher–student developmental relationships, and communicative competence).

Second, structural equation modeling (SEM) was conducted using AMOS to test the hypothesized model, including both direct and mediated paths. SEM was appropriate for this study because it allows the simultaneous estimation of multiple relationships between latent constructs. The mediating role of teacher–student developmental relationships was tested using bias-corrected bootstrapping with 5,000 resamples, which provided 95% confidence intervals for the indirect effects.

Model fit was evaluated using established indices (e.g., RMSEA, CFI, TLI), which indicated acceptable fit following Hu and Bentler’s (1999) recommendations. For all structural paths, we report standardized coefficients (*β*), explained variance (R^2^), and 95% bootstrap confidence intervals, in addition to *p*-values. This approach ensures greater transparency by presenting both the magnitude and the precision of the estimated effects, rather than relying solely on significance testing.

## Results

### Descriptive and correlational analyses

The descriptive statistics for the key variables in the study are presented below in Table 3. The mean score for CLT was 3.41 (SD = 0.64), while form-focused instruction had a lower mean of 2.68 (SD = 0.83). Teacher-students’ developmental relationships had a mean score of 3.64 (SD = 0.70), and communicative competence had a mean of 3.35 (SD = 0.89). These values are based on a sample of 891 participants.

**Table 3:** Descriptive statistics and Pearson’s correlations for key variables. (*N* = 891).

Variable	Mean	SD	1	2	3	4
1. Communicative language teaching (CLT)	3.41	0.64	*0.65*			
2. Form-focused instruction	2.68	0.83	−0.428**	*0.68*		
3. Teacher-students’ developmental relationships	3.64	0.70	0.457**	−0.424**	*0.74*	
4. Communicative competence	3.35	0.89	0.348**	−0.376**	0.512**	*0.85*

*p* < 0.01. Values in italics in the diagonal are Cronbach’s Alphas.

The Pearson’s correlation matrix indicated several significant relationships between the variables. CLT was positively correlated with teacher-students’ developmental relationships (r = 0.457, *p* < 0.01) and communicative competence (r = 0.348, *p* < 0.01). However, CLT was negatively correlated with form-focused instruction (r = −0.428, *p* < 0.01), indicating that higher levels of CLT are associated with lower levels of form-focused instruction.

Form-focused instruction was negatively correlated with both teacher-students’ developmental relationships (r = −0.424, *p* < 0.01) and communicative competence (r = −0.376, *p* < 0.01), suggesting that a greater emphasis on form-focused teaching is associated with lower developmental relationships and communicative competence.

Teacher-students’ developmental relationships were positively correlated with communicative competence (r = 0.512, *p* < 0.01), showing that stronger relationships between teachers and students are associated with higher levels of communicative competence.

These correlations suggest significant relationships between teaching methods, developmental relationships, and communicative competence in the classroom setting.

### Structural equation modeling

#### Model fit

The structural equation model yielded χ^2^(99) = 661.88, *p* < 0.001, with a chi-square to degrees of freedom ratio (CMIN/DF) of 6.69. Although the chi-square statistic was significant, this outcome is common in large samples and does not necessarily indicate poor fit. The RMSEA was 0.080 (90% CI [0.074, 0.086]), with a PCLOSE value of 0.000, suggesting that the model fit was adequate but not close to perfect. Incremental fit indices supported this interpretation, with CFI = 0.917, IFI = 0.917, TLI = 0.900, and NFI = 0.904, all exceeding the 0.90 threshold for acceptable fit. The GFI = 0.922 and AGFI = 0.893 were near recommended cutoffs. The RMR was 0.194, higher than ideal but interpretable given the complexity of the model. Parsimony-adjusted measures (PNFI = 0.746, PCFI = 0.757) indicated a reasonable balance between model fit and simplicity.

Taken together, these indices suggest that the structural model provides an acceptable, though not perfect, representation of the data. Despite some indices indicating only moderate fit, the model adequately captures the complexity of the hypothesized relationships and is sufficiently robust for hypothesis testing.

#### Hypotheses testing

The model explained 43% of the variance in teacher–student developmental relationships (R^2^ = 0.43, 95% CI [0.36, 0.50]) and 39% of the variance in communicative competence (R^2^ = 0.39, 95% CI [0.32, 0.44]).

##### Direct hypotheses

Results supported a significant positive effect of Communicative Language Teaching (CLT) on teacher–student developmental relationships (*β* = 0.63, 95% CI [0.56, 0.69], *p* < 0.001), confirming H1. By contrast, Form-Focused Instruction (FFI) had a significant negative effect on teacher–student developmental relationships (β = −0.19, 95% CI [−0.27, −0.10], *p* < 0.01), contrary to H2. Teacher–student developmental relationships, in turn, strongly predicted communicative competence (β = 0.59, 95% CI [0.50, 0.67], *p* < 0.001), supporting H3. However, the direct effects of CLT (β = 0.05, ns) and FFI (β = −0.00, ns) on communicative competence were not significant.

##### Indirect hypotheses

Bootstrapped mediation analyses showed that CLT predicted communicative competence indirectly via teacher–student developmental relationships (β = 0.37, 95% CI [0.29, 0.44], *p* < 0.001), supporting H4. In contrast, the indirect effect of FFI on communicative competence through TSDR was significant but negative (β = −0.28, 95% CI [−0.42, −0.14], *p* < 0.01), thus not supporting H5, as Table 4 shows.

**Table 4:** Standardized path coefficients, confidence intervals, and significance (AMOS, *N* = 891).

Path	β	95% CI	*p*	Result
Direct effects				
CLT → TSDR	0.63	[0.56, 0.69]	< 0.001	H1 supported
FFI → TSDR	−0.19	[−0.27, −0.10]	0.001	H2 not supported (negative)
TSDR → Communicative Competence	0.59	[0.50, 0.67]	< 0.001	H3 supported
CLT → Communicative competence	0.05	[−0.05, 0.14]	0.417	n.s.
FFI → Communicative competence	−0.00	[−0.08, 0.08]	0.954	n.s.
Indirect effects				
CLT → CC (via TSDR)	0.37	[0.29, 0.44]	< 0.001	H4 supported
FFI → CC (via TSDR)	−0.28	[−0.42, −0.14]	0.001	H5 not supported (negative)
Total effects				
CLT → Communicative competence	0.41	[0.34, 0.49]	< 0.01	Supported
FFI → Communicative competence	−0.25	[−0.44, −0.06]	0.055	Marginal

CLT, Communicative language teaching; FFI, Form-focused instruction; TSDR, Teacher–student developmental relationships; CC, Communicative competence; β, standardized regression coefficient; CI, confidence interval; n.s., not significant.

##### Total effects

Considering both direct and indirect paths, CLT had a significant positive total effect on communicative competence (β = 0.41, 95% CI [0.34, 0.49], *p* < 0.01). The total effect of FFI on communicative competence was negative and only marginally significant (β = −0.25 to −0.44, *p* = 0.055, 95% CI [−0.44, −0.06]).

**Figure 2:**
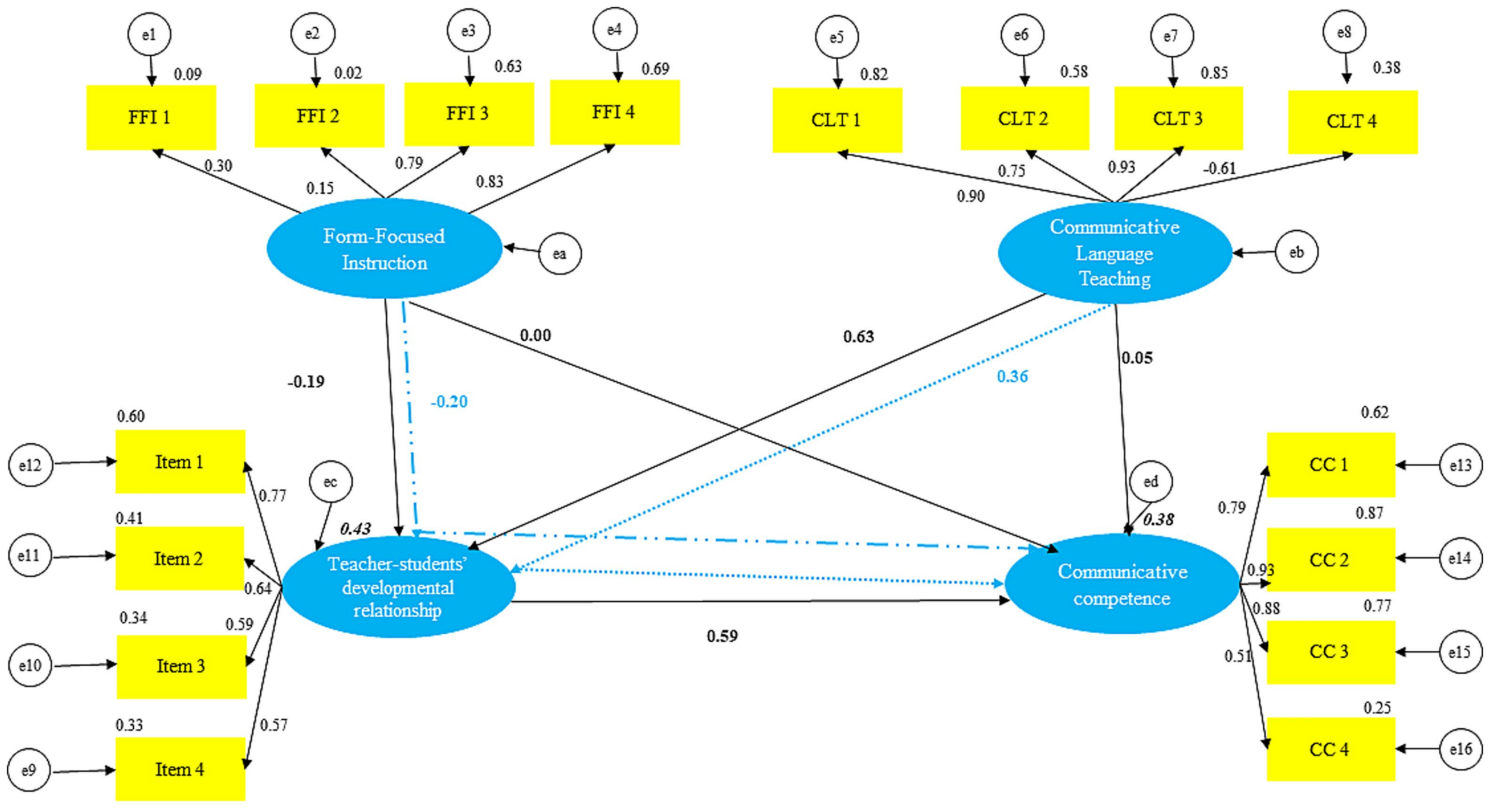
Standardized estimates of the research model. The figure displays direct and indirect pathways among Communicative Language Teaching (CLT), Form-Focused Instruction (FFI), Teacher–Student Developmental Relationships (TSDR), and Communicative Competence (CC). Solid arrows represent significant paths (*p* < 0.05), while dashed arrows indicate non-significant paths. Standardized regression coefficients (*β*) are shown along the arrows.

Figure 2 illustrates the standardized estimates of the structural model, showing the direct and indirect pathways among the study variables. In particular, the figure highlights how form-focused instruction exerts an indirect negative influence on communicative competence through its adverse effect on teacher–student relationships. By contrast, communicative language teaching positively contributes to communicative competence via stronger teacher–student relationships. These results emphasize the central mediating role of teacher–student relationships in transforming instructional practices into either favorable or unfavorable outcomes for students’ language learning.

## Discussion

This study examined the role of Communicative Language Teaching (CLT) and Form-Focused Instruction (FFI) in predicting Chinese EFL learners’ communicative competence, with teacher–student developmental relationships as a mediator. The revised SEM model demonstrated acceptable fit and yielded three key insights: (a) CLT fosters stronger teacher–student relationships, which in turn enhance communicative competence; (b) FFI negatively affects these relationships, reducing its potential to support communicative outcomes; and (c) communicative competence is shaped more by relational mediation than by direct instructional effects.

### Direct hypotheses

The first hypothesis, that CLT would positively predict teacher–student developmental relationships, was supported. This finding confirms that classrooms adopting CLT principles provide more supportive environments, where students feel encouraged to participate and build trust with teachers. Recent studies in Chinese contexts highlight similar benefits of CLT, noting that interactive practices promote motivation and reduce anxiety when coupled with supportive teacher behaviors (Jiang and Paulino, 2024; Liu, 2025). Research in Chinese secondary schools also shows that when teachers emphasize authentic interaction, students report stronger rapport and greater willingness to communicate (Zhu, 2025).

By contrast, the second hypothesis was not supported: FFI negatively predicted teacher–student relationships. This result underscores the limitations of grammar-centered instruction in China, where examination-driven educational traditions often privilege accuracy over interaction. Studies in Sichuan and other provinces have documented that when grammar is emphasized rigidly, students tend to perceive teachers as evaluators rather than facilitators, leading to relational distance (Li and Xu, 2025; Gu, 2025). These findings resonate with our results, suggesting that over-reliance on FFI can undermine the relational foundation necessary for communicative learning.

The third hypothesis, that teacher–student relationships would positively predict communicative competence, was strongly supported. Consistent with both Chinese and international evidence, positive teacher–student rapport fosters student engagement, reduces anxiety, and encourages linguistic risk-taking (Ye and Wang, 2024; Backlund, 2020). Recent Chinese studies also confirm that supportive teacher–student interactions promote learners’ ability to use language strategically and appropriately in varied contexts (Liu, 2025; Wang and Ren, 2025). Our results highlight the centrality of relational quality in determining communicative outcomes, even in classrooms where methodological differences exist.

### Indirect hypotheses

The fourth hypothesis, that CLT would indirectly predict communicative competence through teacher–student relationships, was supported. Although CLT did not exert a significant direct effect on communicative competence, its positive influence emerged clearly when mediated by supportive teacher–student dynamics. This finding aligns with recent evidence showing that CLT’s success in Chinese classrooms depends on reducing student anxiety and fostering a sense of relatedness and motivation (Hu and Wang, 2023; Jiang and Paulino, 2024). In practice, CLT appears to build communicative competence not through the method alone, but through the relational scaffolding it enables.

The fifth hypothesis was not supported: FFI negatively predicted communicative competence through its adverse effect on teacher–student relationships. This suggests that grammar-focused instruction, when dominant, undermines the relational context needed for communicative practice, thereby reducing competence. This result echoes recent Chinese scholarship that warns against overemphasizing accuracy at the expense of interaction, noting that students in exam-oriented contexts often disengage from communicative tasks when they perceive grammar as the central focus (Xu and Wu, 2025; Lu et al., 2025). Our study adds empirical evidence that the relational costs of FFI may outweigh its intended benefits for communicative development.

However, this negative pathway should be interpreted with caution. Rather than implying that FFI inherently undermines relational quality, the data suggest that its adverse effects occur mainly when grammar-focused practices are applied rigidly or dominate instructional time. When integrated flexibly with communicative tasks and delivered within a supportive teacher–student environment, FFI can still serve a valuable complementary role by reinforcing linguistic accuracy and building learners’ confidence in form use. This more nuanced interpretation underscores the conceptual contribution of the present study, which reframes FFI not as an opposing paradigm to CLT but as a resource whose effectiveness depends on the relational and pedagogical context in which it is enacted.

Together, these findings provide a nuanced picture of English teaching in Chinese higher education. CLT offers clear benefits but achieves its impact primarily through fostering positive teacher–student relationships, while FFI, when practiced rigidly, risks damaging those relationships and hindering communication. These results help explain the persistent variability in CLT outcomes reported in China (Zhu, 2025). They also reinforce calls for contextually adapted pedagogies that combine communicative tasks with relationally supportive teaching practices (Li and Xu, 2023; Guo et al., 2023).

These findings must also be interpreted within the broader Chinese sociocultural framework that shapes classroom interaction. Hierarchical teacher roles and collectivist norms often position educators as authority figures rather than facilitators, which can constrain students’ willingness to communicate openly. In addition, exam-oriented pressures place a premium on grammatical accuracy and error avoidance, reinforcing cautious participation and elevating the role of form-focused instruction. Within such a context, supportive teacher–student relationships become essential for humanizing instruction, encouraging risk-taking, and balancing the demands of accuracy and fluency. These sociocultural dynamics likely moderate the pathways observed in the present study, explaining why relational quality emerged as a critical mechanism for communicative development among Chinese EFL learners.

By demonstrating the central mediating role of teacher–student developmental relationships, this study contributes to ongoing debates about language pedagogy in China. It highlights that instructional methods alone are insufficient to enhance communicative competence unless they are embedded in supportive, trust-based classroom dynamics.

### Limitations of the present study and suggestions for future research

This study provides insights into the relationships between communicative language teaching, form-focused instruction, teacher–student developmental relationships, and communicative competence among EFL learners in Sichuan province. Nevertheless, several limitations should be carefully considered when interpreting the findings.

A first limitation is the exclusive reliance on self-reported data. Self-reports inherently involve subjective perceptions and may introduce biases, such as social desirability or inaccurate self-assessment, which can affect the validity of the results. Due to resource and logistical constraints, objective measures such as standardized proficiency tests or direct classroom observations could not be implemented. Future research should combine self-reports with objective assessments to strengthen the robustness of findings. Another limitation concerns the use of English-language instruments. While all participants were proficient English majors, the absence of a formal back-translation or pilot test may have introduced minor linguistic or cultural nuances in item interpretation. Future studies could include bilingual validation to ensure equivalence across languages.

Second, the study employed a cross-sectional design, which restricts the ability to draw causal conclusions. Although structural equation modeling provided insights into directional associations, the mediation effects identified must be interpreted as exploratory. Longitudinal or experimental designs are needed to establish temporal precedence and clarify whether teacher–student relationships indeed mediate the link between instructional approaches and communicative competence.

Third, although the sample was diverse in terms of gender, age, and discipline, it was limited to college students in Sichuan province. This geographical and developmental focus may limit the generalizability of the results. Future studies should include participants from other regions of China and different educational stages to assess whether the observed patterns hold across contexts (Hu, 2002).

A further limitation concerns the instruments employed. The scales for CLT and FFI were adapted from existing frameworks but were not pilot tested prior to use, which may have contributed to the relatively low internal consistency of some measures (*α* < 0.70). Similarly, communicative competence was assessed with single-item indicators for each dimension (grammatical, sociolinguistic, discourse, and strategic competence). While these items showed acceptable reliability, they cannot fully capture the multidimensionality of the construct. Future research should employ validated multi-item instruments and conduct pilot testing to ensure reliability and validity.

In addition, all instruments and instructions were administered in English, with the exception of the Chinese validated version of the engagement scale. This linguistic discrepancy may have influenced participants’ responses and should be considered when interpreting the findings.

Finally, while the study analyzed CLT and FFI simultaneously, it did not address possible interaction effects between them. Future research could examine how integrated approaches, balancing fluency and accuracy, affect both developmental relationships and communicative competence in examination-driven contexts.

### Suggestions for EFL teachers and considerations for curriculum design

The present findings suggest that the effectiveness of Communicative Language Teaching (CLT) in Chinese classrooms depends strongly on the quality of teacher–student relationships. For practitioners, this implies that implementing CLT should not be limited to adopting interactive tasks but must also involve cultivating trust, emotional support, and reciprocal engagement with students. Teachers can create such environments by gradually introducing communicative activities that allow learners to participate actively while feeling supported.

At the same time, the results indicate that Form-Focused Instruction (FFI), when applied in a rigid, correction-centered way, may undermine teacher–student relationships and, in turn, communicative competence. This suggests that grammar teaching is most effective when embedded within communicative contexts and when delivered in ways that do not compromise relational trust. Teachers should aim to provide brief, supportive feedback during or after communicative tasks rather than prioritizing grammar instruction as the central classroom focus.

Curriculum designers and teacher trainers should therefore emphasize strategies that balance fluency and accuracy within a relationally supportive environment. Professional development programs could include modules on how to adapt CLT activities to examination-oriented contexts while maintaining positive teacher–student dynamics. Strengthening these relational aspects appears essential for ensuring that communicative approaches translate into actual improvements in students’ competence.

In addition to classroom practice, leadership within schools plays a critical role in shaping the conditions under which communicative and relational pedagogies can succeed. As highlighted by Mendenhall et al. (2025), visible leadership actions that foster collective teacher efficacy provide a powerful framework for sustaining innovation. Principals and administrators who model collaborative leadership, support teachers’ professional growth, and cultivate a sense of shared purpose can significantly enhance the ability of educators to integrate CLT in ways that foster both communicative competence and strong teacher–student relationships.

Finally, attention must also be paid to teachers themselves. As Perry (2023) argues, non-native English-speaking teachers often face issues of self-perception and equity in professional contexts. Encouraging such teachers to develop self-appreciation and professional confidence can strengthen their capacity to establish supportive classroom relationships. When teachers value their own linguistic and pedagogical contributions, they are better equipped to provide relational scaffolding and to act as credible models of communicative competence for their students.

In summary, the practical implications of this study highlight four priorities for English teaching in China: (1) fostering positive teacher–student relationships as a foundation for communicative learning; (2) embedding grammar instruction within communicative tasks to prevent relational strain; (3) supporting teachers with training and curriculum design that recognize the relational mediation underlying successful CLT implementation; and (4) empowering both school leaders and non-native English-speaking teachers to cultivate professional environments where communicative pedagogy can thrive.

## Conclusion

This study examined the role of Communicative Language Teaching (CLT) and Form-Focused Instruction (FFI) in shaping Chinese EFL learners’ communicative competence, highlighting the mediating role of teacher–student developmental relationships. The findings indicate that CLT does not directly predict communicative competence; instead, its effectiveness is realized through supportive teacher–student relationships. In contrast, FFI showed a negative association with these relationships, which in turn reduced communicative competence. These results underscore that relational dynamics are central to understanding how instructional approaches translate into communicative outcomes.

The study also illustrates the challenges faced by Chinese learners, including examination-driven contexts, limited opportunities for authentic interaction, and cultural traditions that may resist student-centered methods. Teachers who foster positive, supportive relationships with their students can help mitigate these barriers and enhance communicative development.

While the study contributes to ongoing debates on language pedagogy in China, it is not without limitations, particularly the reliance on self-reported data, the cross-sectional design, and the geographically restricted sample. These limitations suggest caution in interpreting the findings and point to the need for more robust, longitudinal, and regionally diverse studies.

Future research should further investigate how CLT and FFI can be integrated in ways that balance fluency and accuracy, and how relational scaffolding can sustain communicative gains over time. In doing so, scholars can contribute to the development of instructional models that are both pedagogically effective and culturally responsive.

## Data Availability

The raw data supporting the conclusions of this article will be made available by the authors, without undue reservation.
